# ST-GICM: A Spatiotemporal Graph Learning Framework with Intrinsic Curiosity for Robust Autonomous Exploration

**DOI:** 10.3390/s26113307

**Published:** 2026-05-22

**Authors:** Linqing He, Weifeng Liu, Wanyu Li

**Affiliations:** College of Electrical and Control Engineering, Shaanxi University of Science and Technology, Xi’an 710021, China; 230611024@sust.edu.cn (L.H.); 230612042@sust.edu.cn (W.L.)

**Keywords:** autonomous exploration, graph neural networks, curiosity-driven learning, spatiotemporal memory, reinforcement learning

## Abstract

With recent advances in deep reinforcement learning (DRL) and graph neural networks (GNNs), graph-based autonomous exploration methods have significantly improved decision-making performance in complex environments. However, under partial observability and sparse-reward conditions, existing methods still struggle with long-horizon decision-making and sustained exploration. To address these challenges, we propose a spatiotemporal graph learning framework, termed ST-GICM, that improves the robustness and efficiency of autonomous exploration by integrating graph-structured encoding, temporal memory, and an intrinsic curiosity mechanism. Specifically, a Graph Attention Network (GAT) and a Spatiotemporal Reasoning Core (STRC) are employed to dynamically encode the viewpoint graph and fuse temporal memory, thereby alleviating perceptual aliasing in graph-based exploration. In addition, an Intrinsic Prediction Module (IPM) is designed to generate intrinsic rewards based on the prediction error of graph-level latent representations, thereby encouraging sustained exploration. Experiments conducted in procedurally generated complex topological environments show that the proposed method outperforms existing baselines in terms of coverage rate, success rate, repeated revisit rate, and oscillation count, while maintaining trajectory costs comparable to those of the baselines. These results demonstrate the effectiveness and superiority of ST-GICM in partially observable environments under sparse-reward conditions.

## 1. Introduction

Autonomous exploration by mobile robots in unknown environments is fundamental to applications such as search and rescue, environmental monitoring, and underground cave exploration. Its core objective is to perceive the environment through onboard sensors, such as LiDAR or RGB-D cameras, and plan a trajectory that covers the entire space with the shortest path or in the least amount of time, without relying on any prior map. Although traditional frontier-based methods have been widely adopted, they often exhibit myopic behavior and struggle to achieve globally efficient exploration in complex large-scale environments.

Although autonomous exploration methods based on deep reinforcement learning (DRL) and graph neural networks (GNNs) have made significant progress in recent years [1,2], existing approaches in complex unknown environments still face two major challenges: long-horizon decision-making under partial observability and sustained progress under sparse-reward conditions. In the field of graph-based exploration, previous studies have shown that explicitly modeling the topological relationships among candidate viewpoints can improve both decision-making efficiency and global awareness in large-scale environments [3,4,5,6,7,8,9]. However, most of these methods rely on current observations or static graph representations for decision-making, making agents prone to perceptual aliasing in complex environments, which in turn leads to repeated revisits and local oscillations, especially in scenarios involving symmetric corridors, repetitive rooms, loop structures, or deep dead ends [10,11,12,13,14]. Meanwhile, extrinsic rewards based on coverage gain rapidly become sparse as exploration proceeds, leaving the policy with insufficient motivation during stages of low information gain, which manifests as exploration stagnation, local wandering, or even premature convergence [15,16,17,18,19]. Although existing intrinsic motivation methods can alleviate the sparse-reward problem to some extent, most of them focus on modeling general state novelty, state influence, or prediction error, without adequately considering the graph-structured decision-making process and topology-level novelty representation in autonomous exploration [16,17,18,19,20]. Therefore, how to jointly model spatial topology, temporal memory, and intrinsic driving mechanisms within a graph-structured exploration framework, so as to simultaneously mitigate perceptual aliasing and exploration stagnation, remains an important problem that urgently needs to be addressed.

To address the above challenges, this paper proposes a spatiotemporal graph learning framework with intrinsic curiosity, termed ST-GICM, for robust autonomous exploration. The main contributions of this work are summarized as follows:

1. We propose a spatiotemporal memory-based exploration framework for discrete decision-making on viewpoint graphs. By combining graph-structured encoding with temporal hidden-state aggregation, the proposed framework improves long-horizon decision-making in partially observable environments.
2. We propose an intrinsic reward mechanism based on the prediction error of graph-level latent representations, which elevates the curiosity signal from general observational novelty to topological novelty, thereby enhancing persistent exploration under sparse-reward conditions.
3. We conduct systematic experiments and ablation studies in complex unknown environments containing dead ends, symmetric mazes, and loop structures, and demonstrate the complementary effects of the spatiotemporal memory and intrinsic motivation mechanisms in reducing ineffective backtracking and improving exploration robustness.

## 2. Related Work

Recent exploration methods have increasingly shifted from hand-crafted geometric heuristics to graph-based decision paradigms. Traditional geometric methods, such as frontier-based exploration and NBVP, remain important baselines, but they rely heavily on local heuristics and manually designed rules, which may lead to repeated search and suboptimal decisions in dead ends, loops, and symmetric layouts [21,22].

Learning-based exploration methods improve decision-making adaptability by incorporating historical observations or learned policies. Memory-enhanced approaches provide temporal cues and partially alleviate myopic behavior, but they can still struggle in environments with severe structural ambiguity, such as deep corridors and symmetric mazes [23,24,25]. In parallel, graph-based exploration methods improve structural modeling, candidate-viewpoint selection, and large-scale coordination by explicitly representing topological relations among viewpoints [7,8,9,26].

Intrinsic motivation has also been introduced to encourage continued exploration when external task rewards become less informative, typically through novelty, prediction error, or action uncertainty [27,28]. However, existing studies generally address geometric decision-making, temporal reasoning, and intrinsic exploration drive separately, rather than within a unified graph-based decision framework. This gap motivates the design of ST-GICM, whose distinction from representative exploration paradigms is summarized in Table 1.

To further clarify the scientific novelty of this work beyond merely combining existing modules, Table 1 summarizes the conceptual differences between representative exploration paradigms and the proposed ST-GICM. The key contribution of ST-GICM is not the isolated use of GAT, recurrent memory, or curiosity, but their task-driven coupling around a viewpoint-graph decision process under partial observability. In particular, the proposed framework treats long-horizon exploration as a unified problem of topological representation, temporal disambiguation, and graph-structured intrinsic guidance, which has not been sufficiently addressed in existing exploration frameworks.

Here, “Graph” denotes whether the decision process is explicitly formulated on a graph structure; “Var. action” denotes whether the policy supports a variable-size candidate action set; and “Topo.-aware” indicates whether the memory or exploration drive is constructed at the topological representation level. As shown in Table 1, ST-GICM is distinguished by the joint use of graph-based decision-making, variable-size graph actions, graph-level temporal memory, and topology-aware curiosity.

**Figure 1:**
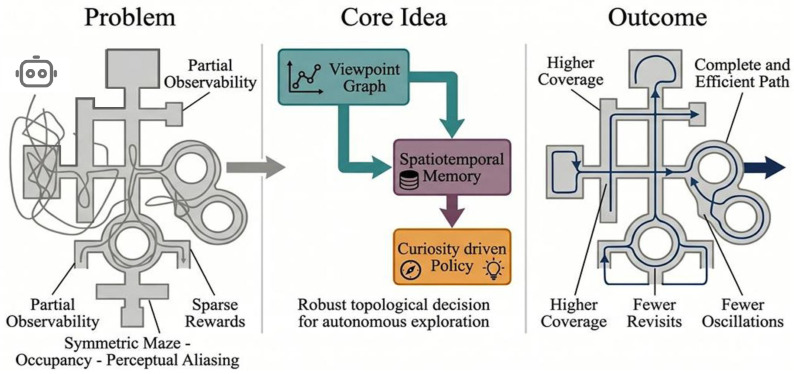
Overview of the proposed ST-GICM framework for 2D high-level autonomous exploration. The large block arrows indicate the logical flow across the three panels; arrows within the Core Idea panel indicate information flow between modules; and arrows in the maze diagrams represent the agent’s exploration trajectories.

Figure 1 illustrates the motivation, framework components, and expected improvements of ST-GICM.

## 3. Environment Representation and Problem Formulation

### 3.1. Mathematical Description of the Exploration Problem

This section establishes a mathematical model for autonomous exploration by a mobile robot in an unknown environment. To reduce the action-space dimensionality in reinforcement learning and improve long-horizon decision-making efficiency, the three-dimensional environment is projected onto a two-dimensional manifold, on which a 2D occupancy grid map is constructed for high-level topological exploration. The underlying three-dimensional physical constraints and obstacle avoidance are handled by a local planner. Consider a mobile robot exploring an unknown bounded two-dimensional space $V\subseteq R^{2}$. The environment is discretized into an occupancy grid map, denoted by M. This map partitions the space into free space $M_{f}$, occupied space $M_{o}$, and unknown space $M_{u}$. Due to the limited field of view (FoV) of the onboard sensors, as well as the effects of environmental occlusion, the robot cannot directly access the global state $s_{t}$ of the environment. Therefore, the exploration process is formulated as a partially observable Markov decision process (POMDP), represented by the seven-tuple:

(1) $$P=\left(S,A,\Omega ,T,O,R,\gamma \right).\;$$

where S denotes the true system state space, including the robot’s dynamic state and the ground-truth map of the environment; A denotes the action space; Ω denotes the observation space; $T(s_{t+1}|s_{t},a_{t})$ denotes the state transition function; $O(o_{t}|s_{t})$ denotes the observation function; $R(s_{t},a_{t})$ denotes the reward function; and γ∈(0,1) denotes the discount factor.

At each discrete time step *t*, the robot receives an observation $o_{t}\in \Omega$ from local sensor measurements and updates its internal map accordingly. Rather than performing high-level decision-making directly in the raw sensor space, the current local observation and map representation are abstracted into a structured viewpoint graph:

(2) $$G_{t}=\left(V_{t},E_{t}\right)\;$$

where the node set $V_{t}$ represents candidate viewpoints in the currently known free space, and the edge set $E_{t}$ represents the reachable connectivity among these viewpoints. This graph is not the environment itself, but rather a topological abstraction used for high-level decision-making.

### 3.2. Viewpoint Graph Representation and Discrete Action Space

On the viewpoint graph $G_{t}=\left(V_{t},E_{t}\right)$, the robot performs discrete decision-making by selecting graph nodes as high-level navigation targets. Let $v_{c}$ denote the graph node currently associated with the robot. Then, the feasible action set at time step *t* is defined as the set of neighboring candidates of the current node:

(3) $$A_{t}=N\left(v_{c}\right)=\left\{v_{j}\in V_{t}|\left(v_{c},v_{j}\right)\in E_{t}\right\}\;$$

That is, an action $a_{t}$ corresponds to selecting a candidate node from the neighborhood of the current node as the high-level target viewpoint for the next time step. The low-level planner then drives the robot toward the target node while continuously updating observations and the map representation during motion. This hierarchical design transforms exploration in continuous space into discrete decision-making on a graph, preserving global structural information while reducing the action complexity of policy learning.

### 3.3. Temporal Hidden State and Policy Definition

Since a single-frame graph observation $G_{t}$ only reflects the current local map state, perceptual aliasing can easily arise in scenarios such as symmetric corridors, repetitive rooms, or loop structures. To support stable long-horizon exploration, this paper introduces a temporal hidden state $h_{t}$ to approximately represent the robot’s internal belief state at time step *t*, which is jointly determined by the current graph observation and historical information.

(4) $$\pi_{\theta }\left(a_{t}|h_{t}\right),\;h_{t}=f\left(G_{t},h_{t-1}\right)$$

where f(·) denotes the temporal state update process that fuses the current graph observation with historical information, and $\pi_{\theta }$ denotes the parameterized policy. Conditioned on the temporal hidden state $h_{t}$, the policy outputs an action probability distribution over the discrete action space $A_{t}$, thereby selecting the next target viewpoint.

The optimization objective of this paper is to maximize the expected cumulative discounted return:

(5) $$J=E\left[\sum_{k=0}^{T}\gamma^{k}r_{t+k}\right]$$

where $r_{t+k}$ denotes the immediate reward at time step t+k, which is designed to encourage the robot to expand the known area, improve exploration completeness, and reduce redundant motion cost. The specific graph representation learning method, temporal reasoning mechanism, and reward construction strategy are introduced in Section 4.

## 4. Method

**Figure 2:**
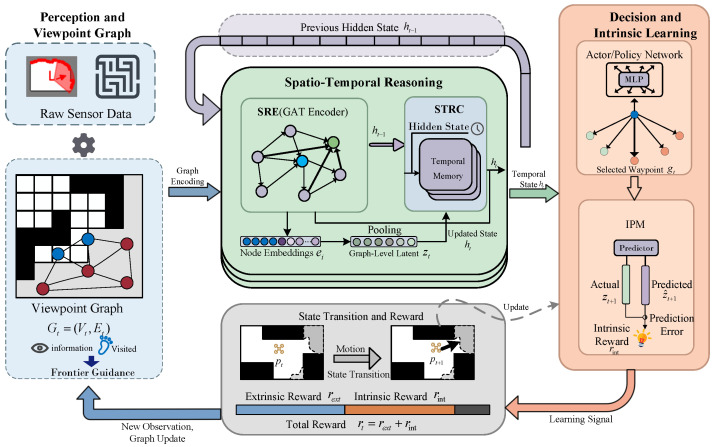
Overall architecture of the proposed ST-GICM framework. The framework consists of three modules: Perception and Viewpoint Graph (blue), Spatio-Temporal Reasoning (green), and Decision and Intrinsic Learning (orange). In the Viewpoint Graph, blue nodes denote informative (frontier) viewpoints and red nodes denote visited viewpoints. Solid arrows indicate the forward data flow between modules; the dashed arrow labeled “Update” represents the feedback loop from the state-transition step back to the Spatio-Temporal Reasoning module. The stacked panels in the Spatio-Temporal Reasoning module illustrate the temporal unrolling of the recurrent reasoning process across multiple time steps.

Following the problem formulation in Section 3, this section presents the proposed ST-GICM framework. As shown in Figure 2, ST-GICM operates on the online-maintained viewpoint graph $G_{t}=\left(V_{t},E_{t}\right)$ and integrates three modules: the Spatial Representation Encoder (SRE), the Spatio-Temporal Reasoning Core (STRC), and the Intrinsic Prediction Module (IPM). The SRE extracts node-level and graph-level representations from the current graph, the STRC updates the temporal hidden state $h_{t}$ using historical information, and the IPM generates an intrinsic reward from graph-level latent prediction error. Based on the temporal state and neighboring candidate viewpoints, the policy selects the next target viewpoint for execution by the low-level planner. The following subsections describe these components in detail.

### 4.1. Viewpoint Graph Construction

To transform the exploration problem in continuous space into a high-level discrete decision-making problem, this paper dynamically maintains a viewpoint graph $G_{t}=\left(V_{t},E_{t}\right)$ at time step *t* based on the occupancy grid map $M_{t}$ constructed online in real time. Here, the node set $V_{t}$ represents candidate observation locations in the known free space, and the edge set $E_{t}$ describes the reachable connectivity among nodes.

#### 4.1.1. Node Generation and Incremental Update

Let $M_{free}$ denote the currently known free space. A uniform sampling strategy is adopted to generate candidate nodes within $M_{free}$. Specifically, regular sampling is performed over the free space at a resolution of δ, and only those nodes satisfying the safety-clearance constraint are retained:

(6) $$d\left(v_{i},M_{obs}\right)>d_{safe}$$

where d(·) denotes the Euclidean distance to the nearest obstacle, which can be obtained from the ESDF map.

To ensure computational efficiency in real time, the viewpoint graph is updated incrementally. When the robot detects newly discovered free regions, additional sample points are generated only within those newly unlocked areas and added to the set $V_{t}$. Meanwhile, if an existing node is determined to be occupied or no longer satisfies the safety-clearance requirement, it is removed from $V_{t}$. This mechanism avoids the computational redundancy caused by rebuilding the graph from scratch at every step.

#### 4.1.2. Edge Connection and Reachability Check

For any newly added node $v_{i}\in V_{t}$, edges are established within its neighborhood to form a sparse roadmap. A *k*-nearest-neighbor connection strategy is adopted. Specifically, when $v_{j}$ belongs to the *k* nearest neighbors of $v_{i}$ and the distance satisfies $∥p_{i}-p_{j}∥\leq r_{max}$, the edge $\left(v_{i},v_{j}\right)$ is considered for inclusion in the set $E_{t}$. To ensure collision-free connectivity and reachability, each candidate edge is subjected to a fast collision check, for example, by sampling points along the connecting line segment. If the candidate edge passes the test, it is retained and its weight is set as the Euclidean distance,

(7) $$w_{ij}=∥p_{i}-p_{j}∥$$

#### 4.1.3. Current Node Association

Since the high-level policy performs discrete decision-making on the graph, the robot’s current continuous pose $x_{t}$ must be associated with a graph node. The current node $v_{c}$ is defined as the reachable graph node closest to the robot position $p(x_{t})$:

(8) $$v_{c}=\arg\min_{v_{i}\in V_{t}}∥p\left(x_{t}\right)-p_{i}∥\;s.t.\;\mathrm{reachable}\left(x_{t}\to v_{i}\right)$$

where reachable(·) denotes collision-free straight-line reachability in the currently known map.

### 4.2. Node Feature Definition and Information Gain Computation

**Figure 3:**
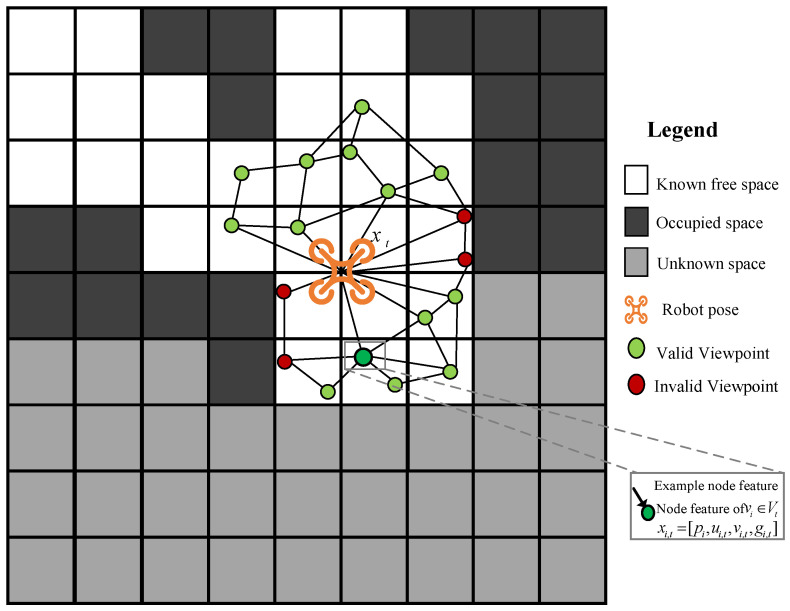
Illustration of viewpoint graph construction and node feature definition. Candidate viewpoints are incrementally sampled in the known free space and connected according to reachability constraints to form the viewpoint graph. Each node is associated with exploration-related features, including exploration gain, visitation status, and frontier-guidance information, which are used as inputs to the graph encoder.

In the viewpoint graph $G_{t}=\left(V_{t},E_{t}\right)$, each node $v_{i}\in V_{t}$ corresponds to a candidate viewpoint position $p_{i}$. To inject geometric and exploration-related information into the graph encoder, a feature vector is constructed for each node:

(9) $$x_{i,t}=\left[p_{i},u_{i,t},v_{i,t},g_{i,t}\right]$$

where $u_{i,t}$ denotes the potential exploration gain of the viewpoint, $v_{i,t}$ describes its historical visitation status, and $g_{i,t}$ provides guidance toward the global frontier. The overall process of viewpoint graph construction and feature definition is illustrated in Figure 3.

#### 4.2.1. Exploration Gain $u_{i,t}$

Let $M_{t}$ denote the current occupancy grid map, and let $U_{t}$ denote the set of unknown grid cells in the map. For each candidate viewpoint $p_{i}$, its visible grid-cell set, denoted by $\mathrm{Vis}(p_{i})$, is obtained through ray casting or visibility checking. The viewpoint gain is defined as the number of unknown grid cells observable from that viewpoint:

(10) $$u_{i,t}=\left|\mathrm{Vis}\left(p_{i}\right)\cap U_{t}\right|$$

To avoid repeated counting, an incremental form based on newly visible unknown cells can also be adopted, where only the additional unknown coverage introduced relative to the previous decision cycle is counted.

#### 4.2.2. Visitation Status $v_{i,t}$

To explicitly encode visited/unvisited information and reduce local oscillation, a visitation flag is maintained for each node. Let $p(x_{t})$ denote the robot position at time step *t*. A node $v_{i}$ is regarded as visited when

(11) $$∥p\left(x_{t}\right)-p_{i}∥\leq d_{visit}$$

The visitation status can then be defined as a binary variable:

(12) $$v_{i,t}=I\;\left[\mathrm{visited}(v_{i})\right]$$

It can also be extended to finer-grained representations, such as the visit count or the elapsed time since the last visit, to enhance temporal distinguishability.

#### 4.2.3. Guidance Feature $g_{i,t}$

Relying only on local gain may cause the policy to behave myopically in long corridors or large open spaces. Therefore, this paper introduces guidance information toward the global frontier. Let $F_{t}$ denote the set of frontiers in the current map, i.e., the boundary between unknown and known free space. The shortest-path cost from node $v_{i}$ to the frontier, denoted by $d_{i,t}^{F}$, is defined as the shortest path length on the graph $G_{t}$ (or on the occupancy grid using $A^{*}$):

(13) $$d_{i,t}^{F}=\min_{f\in F_{t}}\mathrm{Dist}\left(p_{i},f\right)$$

Furthermore, to provide directional guidance, let $f^{*}$ denote the frontier point (or frontier cluster center) that attains the minimum in (13). The guidance vector is then defined as

(14) $$g_{i,t}=\frac{f^{*}-p_{i}}{∥f^{*}-p_{i}∥}$$

In this way, the policy can obtain global guidance toward the nearest effective frontier region while learning local decision-making behaviors.

### 4.3. Spatial Representation Encoder (SRE)

The core of the Spatial Representation Encoder (SRE) is to encode the viewpoint graph using a Graph Attention Network (GAT). Given the node features $\left\{x_{i,t}\right\}$, a GAT is employed to encode each node and produce node embeddings $\left\{e_{i,t}\right\}$. For node $v_{i}$, its embedding is updated as

(15) $$e_{i,t}=\sigma \left(\sum_{j\in N\left(v_{i}\right)\cup \left\{v_{i}\right\}}\alpha_{ij,t}Wx_{j,t}\right)$$

where W is a linear transformation matrix, σ(·) is a nonlinear activation function, and $\alpha_{ij,t}$ is the attention weight that characterizes the contribution of neighboring node $v_{j}$ to node $v_{i}$. It is computed as

(16) $$\alpha_{ij,t}={\mathrm{softmax}}_{j}\;\left(\mathrm{LeakyReLU}\;\left(a^{⊤}\left[Wx_{i,t}\;∥\;Wx_{j,t}\right]\right)\right)$$

The above attention mechanism adaptively selects more informative neighborhood information according to the geometric and exploration-related attributes of the nodes, enabling the node embeddings to capture both local structural patterns and semantic context.

In addition to node-level representations, a global aggregation operation is further applied to the node embeddings to obtain a graph-level representation $z_{t}$, which serves as a macroscopic summary of the overall exploration state for the temporal reasoning module:

(17) $$z_{t}=\mathrm{Pool}\;\left(\{e_{i,t}|v_{i}\in V_{t}\}\right)$$

where Pool(·) denotes a global pooling operation, such as mean pooling. Finally, the graph-level representation $z_{t}$ is fed into the STRC for temporal memory aggregation, while the node-level embeddings $\left\{e_{i,t}\right\}$ are used by the high-level policy to score and select actions over neighboring candidates.

### 4.4. Spatio-Temporal Reasoning Core (STRC)

**Figure 4:**
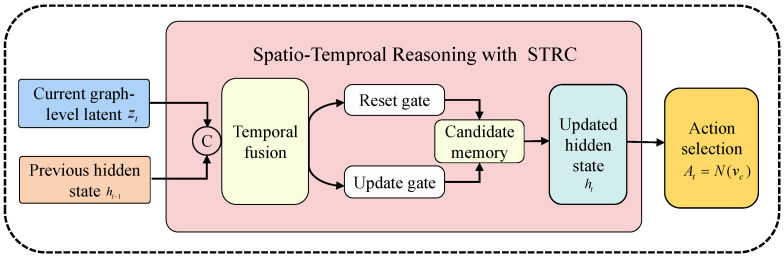
Structure of the Spatio-Temporal Reasoning Core (STRC). The colors are used to visually distinguish the functional components of the STRC module. The module takes the current graph-level representation $z_{t}$ and the previous hidden state $h_{t-1}$ as inputs, updates the temporal memory through recurrent reasoning, and outputs the current hidden state $h_{t}$ for downstream policy learning and intrinsic prediction.

Making decisions solely based on the current spatial embedding $z_{t}$ corresponds to a reactive policy. In environments with highly similar structures, such as symmetric corridors or repetitive rooms, different physical locations may produce indistinguishable local observations, which can in turn lead to in-place oscillation or repeated revisits. To address this issue, this paper introduces a Spatio-Temporal Reasoning Core (STRC) to maintain memory of the exploration trajectory, as shown in Figure 4.

Specifically, the STRC adopts a recurrent neural network based on a Gated Recurrent Unit (GRU). It takes the current spatial embedding $z_{t}$ and the previous memory state $h_{t-1}$ as inputs to update the current latent belief state $h_{t}$: STRC is not intended to replace the standard GRU as a new recurrent unit. Rather, it is a task-specific temporal reasoning module that uses a GRU backbone to update the graph-level hidden state from successive viewpoint-graph observations. Its main distinction from a standard GRU lies in the representation and decision context: STRC operates on graph-level latent representations produced by the SRE and couples the updated hidden state with graph-based action selection and intrinsic prediction. Therefore, STRC performs temporal fusion at the viewpoint-graph representation level, rather than serving as a generic recurrent module over raw observations or fixed-dimensional state vectors.

(18) $$h_{t}=f_{\mathrm{STRC}}\left(z_{t},h_{t-1};\theta_{\mathrm{mem}}\right)$$

By aggregating historical information along the temporal dimension, the hidden state $h_{t}$ can effectively encode the contextual information of the robot’s exploration trajectory. The policy network then makes decisions based on $h_{t}$, enabling it to distinguish geometrically similar but topologically different regions and thereby support non-myopic long-horizon path planning.

### 4.5. Intrinsic Prediction Module (IPM)

In large-scale complex environments, when frontier regions are sparsely distributed, extrinsic rewards based on coverage gain often approach zero, causing deep reinforcement learning to face the sparse-reward challenge. Inspired by curiosity-driven learning mechanisms, this paper proposes an Intrinsic Prediction Module (IPM). The IPM includes a forward dynamics model that aims to learn the state transition pattern of the environment in the latent space. Specifically, given the current historical state $h_{t}$ and the action $a_{t}$ to be taken, the model predicts the spatial feature at the next time step, denoted by ${\hat{z}}_{t+1}$:

(19) $${\hat{z}}_{t+1}=f_{\mathrm{fwd}}\left(h_{t},a_{t};\phi_{\mathrm{IPM}}\right)$$

The discrepancy between the predicted value and the true observation $z_{t+1}$ is defined as the intrinsic curiosity reward $r_{\mathrm{int},t}$:

(20) $$r_{\mathrm{int},t}=\eta \cdot {∥{\hat{z}}_{t+1}-z_{t+1}∥}^{2}$$

where η is a scaling coefficient. A large prediction error usually indicates that the robot is currently in a novel or complex region that has not yet been well captured by the dynamics model. Therefore, maximizing this intrinsic reward encourages the agent to actively explore regions with high epistemic uncertainty even when extrinsic reward signals are absent.

The final composite reward function $r_{t}$ is constructed as a weighted combination of the extrinsic coverage reward $r_{\mathrm{ext},t}$, the intrinsic curiosity reward $r_{\mathrm{int},t}$, and the motion cost $c_{\mathrm{move}}$:

(21) $$r_{t}=r_{\mathrm{ext},t}+\beta \cdot r_{\mathrm{int},t}-\lambda \cdot c_{\mathrm{move}}$$

The extrinsic coverage reward $r_{\mathrm{ext},t}$ is used to characterize the new information gain brought by exploration. Let $U_{t}$ denote the set of unknown grid cells in the occupancy grid map at time step *t*. Then, the extrinsic reward is defined as the reduction in unknown grid cells within one decision cycle:

(22) $$r_{\mathrm{ext},t}=\left|U_{t-1}\setminus U_{t}\right|$$

This definition directly reflects the newly covered area of the environment during the current decision cycle and avoids repeated counting for previously observed regions. If an area-based formulation is adopted, it is sufficient to multiply this quantity by the grid-cell area constant.

The motion cost $c_{\mathrm{move}}$ is introduced to penalize excessively long displacements and thereby improve exploration efficiency. It is defined as the path length from the current node $v_{c}$ to the target node $a_{t}$, which can be computed either from the shortest path on the viewpoint graph or from the actual trajectory executed by the low-level planner. Taking the graph shortest path as an example:

(23) $$c_{\mathrm{move}}={\mathrm{Dist}}_{G_{t}}\left(v_{c},a_{t}\right)$$

where ${\mathrm{Dist}}_{G_{t}}\left(\cdot \right)$ denotes the shortest-path length on $G_{t}$ computed based on edge weights, such as Euclidean distance or $A^{*}$ cost.

### 4.6. Training Objective and Discrete Soft Actor–Critic

This paper adopts the maximum-entropy Soft Actor–Critic (SAC) algorithm for end-to-end policy learning. Both the actor network $\pi (a_{t}|h_{t})$ and the critic network $Q(h_{t},a_{t})$ take the spatiotemporal hidden state $h_{t}$ as the main conditional input. For the discrete candidate actions in the neighborhood of the current node, the policy network further scores each candidate by combining the candidate node embedding and relative positional information, and then normalizes the scores to obtain an action distribution. The policy objective is given by

(24) $$J\left(\pi \right)=\sum_{t=0}^{T}E_{\left(h_{t},a_{t}\right)∼\rho_{\pi }}\left[r\left(h_{t},a_{t}\right)+\alpha \;H\;\left(\pi (\cdot |h_{t})\right)\right]$$

where H(·) denotes the entropy of the policy, and α is the temperature parameter used to dynamically balance the relative weights of the entropy term and the reward term.

It should be emphasized that the high-level action in this work is discrete: the next target node is selected from the neighborhood of the current node $v_{c}$,

(25) $$a_{t}\in A_{t}=N\left(v_{c}\right)$$

Since the high-level action is a discrete choice over the neighboring candidate set $A_{t}=N\left(v_{c}\right)$, the policy network scores each neighboring candidate individually and normalizes the scores within the neighborhood. Specifically, let $h_{t}$ denote the temporal hidden state produced by the STRC, and let $\left\{e_{i,t}\right\}$ denote the node embeddings produced by the SRE. For any candidate action $a_{t}=v_{j}\in N\left(v_{c}\right)$, we construct the candidate action representation as

(26) $$\psi_{j,t}=\left[h_{t},e_{c,t},e_{j,t},\left(p_{j}-p_{c}\right)\right]$$

and compute its logit using a lightweight scoring function implemented by an MLP:

(27) $$s_{j,t}=f_{\theta }\left(\psi_{j,t}\right)$$

The critic uses the same candidate-conditioned action representation $\psi_{j,t}$ to evaluate $Q(h_{t},a_{t}=v_{j})$, so each action is represented by its corresponding graph node and relative geometry rather than by a fixed discrete identifier. This design allows the actor and critic to handle dynamically changing candidate sets across different viewpoint graphs. A mask is then applied over the neighboring set, followed by a softmax operation to obtain the action distribution:

(28) $$\pi_{\theta }\left(a_{t}=v_{j}|h_{t}\right)=\frac{\exp(s_{j,t})}{\sum_{v_{k}\in N\left(v_{c}\right)}\exp\left(s_{k,t}\right)},\;v_{j}\in N\left(v_{c}\right)$$

This parameterization explicitly exploits both the temporal state $h_{t}$ and the neighborhood candidate features/embeddings, enabling the policy to produce a stable probability distribution over action sets of variable size, which is consistent with the training objective of discrete SAC. Therefore, $\pi_{\theta }\left(a_{t}|h_{t}\right)$ outputs a categorical distribution over the variable action set $A_{t}$. In implementation, an action mask is applied to all non-neighbor candidates so that the policy is normalized only over $A_{t}$, thereby adapting to variable candidate sets under dynamic graph structures.

Under this discrete setting, the soft value can be written as the expectation over the feasible action set:

(29) $$V_{\bar{\phi }}\left(h_{t}\right)=\sum_{a\in A_{t}}\pi_{\theta }\left(a|h_{t}\right)\left(\min_{i\in \{1,2\}}Q_{{\bar{\phi }}_{i}}\left(h_{t},a\right)-\alpha \log\pi_{\theta }\left(a|h_{t}\right)\right)$$

Based on this, the Bellman target for the critic is constructed as

(30) $$y_{t}=r_{t}+\gamma V_{\bar{\phi }}\left(h_{t+1}\right)$$

where double Q-networks are adopted and the minimum of the two estimates is used to alleviate overestimation, and $\bar{\phi }$ denotes the target-network parameters updated by Polyak averaging. The critic is updated by minimizing the mean squared error:

(31) $$J_{Q}\left(\phi_{i}\right)=E_{D}\left[\frac{1}{2}{\left(Q_{\phi_{i}}\left(h_{t},a_{t}\right)-y_{t}\right)}^{2}\right],\;i\in \left\{1,2\right\}$$

The update objective of the actor is to maximize the trade-off between the soft Q-value and entropy regularization. Its equivalent minimization form is given by

(32) $$\begin{array}{cc} J_{\pi }\left(\theta \right)=E_{D} & [\sum_{a\in A_{t}}\pi_{\theta }\left(a|h_{t}\right)\\ \; & \cdot (\alpha \log\pi_{\theta }\left(a|h_{t}\right)-\min_{i}Q_{\phi_{i}}\left(h_{t},a\right))] \end{array}$$

Since the action space is discrete, the above expectation can be computed directly by summing over $A_{t}$, without requiring the reparameterization trick used in SAC for continuous actions.

To make the policy output executable within a simulated robot navigation pipeline, a hierarchical planning architecture is adopted for system integration. ST-GICM serves as the global planning layer and outputs a coarse-grained target waypoint $p_{\mathrm{goal}}$. At the lower level, a gradient-based local planner is integrated to generate a smooth, collision-free, and dynamically feasible continuous trajectory in the constructed local Euclidean Signed Distance Field (ESDF) map, and then sends it to the controller for execution. This hierarchical design combines the long-horizon decision-making capability of reinforcement learning with the advantages of traditional trajectory optimization in local obstacle avoidance and smooth control, thereby improving the overall stability and system-level compatibility of the exploration framework in simulation.

At the execution level, a hierarchical decision-making mechanism is adopted. The high-level policy does not directly output continuous control commands; instead, it selects a target viewpoint from the neighboring candidate set of the current viewpoint node, denoted by $a_{t}$. Given this target, the low-level local planner generates a collision-free trajectory satisfying motion constraints based on the current ESDF map and drives the robot toward the target viewpoint. In this paper, “selecting a target viewpoint and completing one local execution” is defined as one high-level decision cycle. When the robot successfully reaches the selected target viewpoint, or when the local planning execution fails or is interrupted, the current decision cycle terminates. The system then updates the local map based on the robot’s actual reached position, reconstructs the viewpoint graph, and generates the next state $s_{t+1}$.

Accordingly, the reward $r_{t}$ is computed uniformly at the end of the execution cycle. It consists of the coverage increment before and after execution, the graph-level intrinsic reward, and the trajectory execution cost. When local planning fails, it is treated as an unsuccessfully executed high-level action, and only the corresponding cost penalty is retained, without producing any positive coverage gain.

## 5. Experiments and Results

### 5.1. Experimental Environment and Evaluation Baselines

This section evaluates the performance of ST-GICM on 100 randomly generated 2D occupancy grid maps. The simulation environments contain typical topological elements such as corridors and loop structures. A hierarchical planning architecture is adopted: the high-level policy performs discrete decision-making on the online viewpoint graph, while the low-level planner generates smooth trajectories based on the ESDF. A decision cycle is triggered when the robot reaches the target viewpoint or when the local planning process is interrupted.

To improve reproducibility without overloading the main text, the key implementation settings of ST-GICM are summarized in Table 2. The environment and sensor settings are kept consistent with the experimental setup described above.

Since the training maps are procedurally generated online, the training set is not a fixed finite collection of environments. Other low-level settings, including reward normalization and model size, were kept fixed across all experiments.

The main quantitative evaluation is conducted in procedurally generated 2D occupancy-grid environments, while the system-level validation is performed in ROS/Gazebo (ROS Noetic and Gazebo, Open Source Robotics Foundation, Mountain View, CA, USA) simulation. The robot is modeled as a planar mobile platform, and ST-GICM outputs waypoint-level targets rather than low-level control commands. The local planner is responsible for trajectory generation, collision avoidance, and waypoint execution, thereby handling low-level motion feasibility during simulation. The robot is equipped with a simulated range sensor with a horizontal field of view of 120° and a sensing range of 10 m. The occupancy grid map has a resolution of 0.4 m, and viewpoint graph nodes are sampled at a resolution of 4.0 m.

To evaluate the proposed method, four representative baselines are selected for comparison: the greedy frontier-based method Nearest Frontier, the RRT-sampling-based geometric method NBVP, the utility-weighted frontier strategy UW, and the memory-enhanced learning-based framework MARVEL. The selected baselines cover representative exploration paradigms, including frontier-based geometric exploration, sampling-based next-best-view planning, utility-weighted frontier selection, and learning-based graph exploration. Specifically, Nearest Frontier and Utility-Weighted represent widely used frontier-based strategies, NBVP represents sampling-based geometric exploration, and MARVEL represents a memory-enhanced learning-based exploration framework. In addition to these external baselines, the ablation variants Base (GAT), +STRC, and +IPM serve as controlled graph-learning comparisons under the same graph representation and training protocol. Although these variants are not substitutes for independent external baselines, they help isolate the effects of temporal memory and intrinsic prediction while reducing confounding factors introduced by implementation differences.

The evaluation metrics include final coverage rate, cumulative trajectory length, repeated revisit rate, and the number of local oscillations. A trial is considered successful if the robot reaches 90% coverage within the preset time limit. In addition to success rate, the metrics also include the cumulative path length of successful trials, denoted by Dist@90. All results are averaged over multiple runs under identical physical parameters and are reported as mean ± standard deviation. In Table 3, “Trajectory (m)” denotes the average cumulative trajectory length over all evaluation trials until termination. In Table 4, “Dist@90” denotes the cumulative path length when 90% coverage is reached and is computed only over successful trials.

To further quantify repeated and unstable exploration behaviors, we explicitly define the revisit ratio and oscillation count as follows. Let $v_{t}$ denote the graph node associated with the robot at the end of the *t*-th high-level decision cycle, and let $V_{\mathrm{visited}}^{t-1}$ denote the set of graph nodes visited before step *t*.

The revisit ratio $R_{\mathrm{revisit}}$ measures the proportion of decision cycles in which the robot reaches a previously visited graph node:

(33) $$R_{\mathrm{revisit}}=\frac{1}{T}\sum_{t=1}^{T}I\left(v_{t}\in V_{\mathrm{visited}}^{t-1}\right),\;$$

where *T* is the total number of high-level decision cycles in an episode, and I(·) is the indicator function. A lower revisit ratio indicates fewer redundant visits to already explored topological regions.

The oscillation count $C_{\mathrm{osc}}$ measures short-term back-and-forth behavior between neighboring viewpoints. In this work, an oscillation is counted when the robot returns to the same graph node after a two-step interval, corresponding to an A→B→A pattern:

(34) $$C_{\mathrm{osc}}=\sum_{t=3}^{T}I\left(v_{t}=v_{t-2},\;v_{t}\neq v_{t-1}\right).\;$$

A lower oscillation count indicates more coherent and stable long-horizon exploration behavior. Both metrics are computed from the high-level graph-node sequence rather than from low-level controller commands.

### 5.2. Comprehensive Exploration Performance Comparison

To validate the overall effectiveness of ST-GICM, all compared methods were quantitatively evaluated on the aforementioned 100 test maps, and the statistical results are presented in Table 3.

To further assess the statistical reliability of the improvements, we additionally computed 95% confidence intervals over the 100 test maps. For the coverage rate, ST-GICM achieves 92.7±1.23% in terms of 95% CI, while MARVEL achieves 80.1±3.29%. For the revisit ratio, ST-GICM obtains 0.097±0.010, compared with 0.161±0.038 for MARVEL. For the oscillation count, ST-GICM obtains 5.3±0.92, compared with 11.8±4.86 for MARVEL. These confidence intervals support the statistical reliability of the observed improvements in coverage and behavioral stability.

As shown in Table 3, ST-GICM achieves the best overall performance on most key metrics. Nearest Frontier and NBVP perform poorly in complex dead-end and loop structures, resulting in low coverage rates of 61.5–74.7%. Although Nearest Frontier has a relatively low trajectory cost, it shows a success rate of only 32.0%, together with high repeated revisit and oscillation counts. NBVP improves coverage, but at the cost of substantial path redundancy, with an average trajectory length exceeding 1000 m.

MARVEL improves the success rate to 62.0% through graph-structured policy learning, but still exhibits relatively large coverage variance and unstable behavior in some complex environments.

In contrast, ST-GICM reaches an average coverage rate of 92.7% and a success rate of 69.0%, while maintaining a trajectory length comparable to that of MARVEL (759.66 m). Compared with MARVEL, the success-rate improvement from 62.0% to 69.0% is moderate. More notably, ST-GICM achieves the lowest repeated revisit rate and oscillation count, indicating improved behavioral stability and reduced redundant exploration.

### 5.3. Qualitative Analysis in Complex Constrained Scenarios

**Figure 5:**
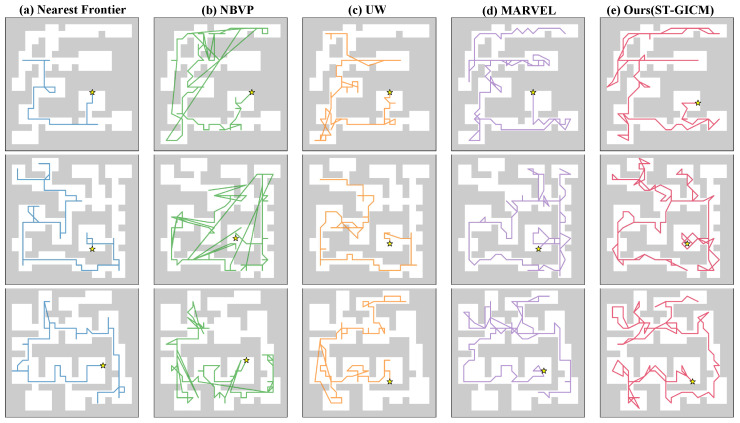
Qualitative comparison of exploration trajectories in three complex topological scenarios: long corridor dead end, symmetric maze, and central loop. The star marker indicates the starting position, and the different colored lines represent the exploration trajectories generated by different methods.

To qualitatively analyze the behaviors of different algorithms in challenging environments, this subsection selects three representative complex topological scenarios, namely a long corridor dead end, a symmetric maze, and a central loop, as shown in Figure 5.

As shown in Figure 5, traditional heuristic and sampling-based algorithms such as Nearest Frontier and NBVP exhibit clear myopic behavior in narrow corridors. In Figure 5a,b, these methods often terminate exploration prematurely due to limited local field of view or failures in viewpoint sampling, leaving large unexplored regions.

MARVEL improves the overall coverage range, but still exhibits unstable behavior in locally constrained spaces. In the deep dead end of Map 1 and the symmetric maze of Map 2, its trajectories show frequent overlaps and crossings, indicating severe perceptual aliasing and repeated local oscillations. This observation is consistent with the high oscillation counts reported in Table 3.

In contrast, ST-GICM produces more coherent exploration trajectories. In the long-corridor dead end of Map 1, it avoids redundant local backtracking, while in the complex maze of Map 2 and the central loop of Map 3, it more effectively avoids previously explored dead ends and continues advancing toward unknown frontier regions. These qualitative results further demonstrate the effectiveness of spatiotemporal memory and intrinsic motivation in improving long-horizon exploration.

### 5.4. Ablation Study on Core Mechanisms

To verify the independent contributions of the Spatio-Temporal Reasoning Core (STRC) and the Intrinsic Prediction Module (IPM) to the overall system performance, this subsection conducts an ablation study using a memoryless Graph Attention Network, denoted as Base (GAT), as the reference model. This ablation base is an internal variant of ST-GICM and is distinct from the external baseline MARVEL. The results are summarized in Table 4.

**Figure 6:**
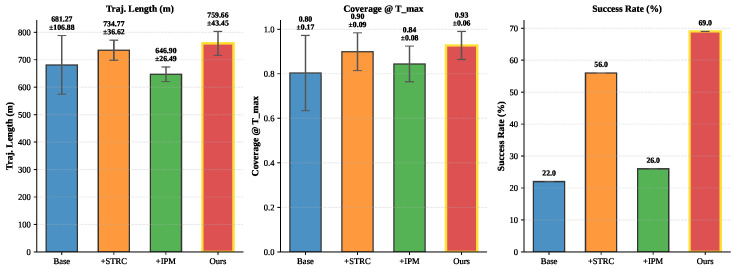
Ablation comparison of different ST-GICM variants.

The experimental results show that introducing STRC leads to significant improvements in both coverage rate and success rate, confirming the central role of the temporal memory mechanism in alleviating perceptual aliasing and enhancing the robustness of long-horizon decision-making. Introducing IPM alone can maintain a certain level of exploration drive when extrinsic rewards become sparse, but its performance gains in complex topologies are relatively limited. The full model achieves the best performance across all evaluation metrics, demonstrating the strong complementarity between STRC and IPM, as further illustrated in Figure 6.

**Figure 7:**
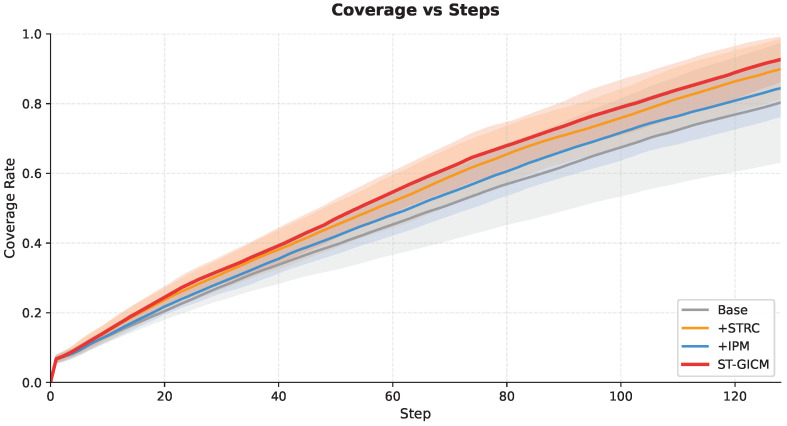
Coverage curves of different ablation variants over decision steps. The shaded regions indicate the standard deviation across test episodes.

Figure 7 presents the average coverage growth curves of different model variants on the test set. The results show that, in the early stage of exploration, the differences in growth slopes among the models are relatively small, since the environment contains a large amount of readily accessible extrinsic rewards. However, as exploration proceeds (after approximately 60 steps), the extrinsic gain gradually becomes sparse, and the coverage curves of the ablation variants without intrinsic motivation (i.e., Base (GAT) and +STRC) begin to flatten, exhibiting evident exploration stagnation.

In contrast, the variants equipped with IPM (i.e., +IPM and the full ST-GICM model) maintain more sustained coverage growth in the middle and later stages of the task. Among them, the full ST-GICM model achieves the best overall performance in both final coverage and the stability of coverage growth, which further supports the complementary roles of STRC and IPM.

### 5.5. Sensitivity Analysis

To examine the stability of ST-GICM under different parameter settings, we conducted a one-factor-at-a-time sensitivity analysis on three key parameters: the intrinsic reward weight β, the graph-node resolution $\delta_{g}$, and the hidden dimension $d_{h}$, which controls the memory capacity of the STRC module. For each group, only the target parameter was changed, while the remaining parameters were kept at their default values, i.e., β=0.1, $\delta_{g}=4.0$ m, and $d_{h}=256$. The experiments were conducted over 30 test episodes using the same evaluation protocol.

As shown in Table 5, ST-GICM remains stable under moderate variations of the intrinsic reward weight. When β varies from 0 to 0.2, the coverage rate remains within 90.51–91.79%, and the oscillation count stays around five. This indicates that the method is not overly sensitive to the exact intrinsic reward weight within the tested range.

For the graph-node resolution, the coverage rate remains above 91% for all tested settings. A finer graph resolution slightly improves coverage, whereas a coarser resolution leads to a mild increase in oscillation count. This suggests that graph resolution affects the balance between topological fidelity and decision complexity.

For the temporal memory size, increasing the STRC hidden dimension from 128 to 256 reduces the oscillation count and improves coverage. Further increasing the dimension to 512 brings limited additional improvement. Overall, the sensitivity results indicate that ST-GICM is not overly sensitive to moderate variations in these key parameters, while maintaining stable exploration performance and comparable decision time.

### 5.6. ROS-Based System-Level Simulation Validation

To verify the system-level feasibility of the proposed algorithm within a standard robot middleware (ROS), system-level closed-loop experiments were conducted in a ROS-based simulation environment. This subsection focuses on evaluating the operational stability of ST-GICM under the coupling of online mapping, viewpoint decision-making, and low-level navigation.

To complement the qualitative trajectory visualization, we further report system-level indicators from representative ROS/Gazebo simulation runs. These indicators are used to evaluate the waypoint-level execution stability and runtime behavior of the simulated navigation pipeline. The ROS/Gazebo runtime measurements were collected on a desktop computer equipped with an Intel Core i7-9700F CPU (Intel Corporation, Santa Clara, CA, USA), an NVIDIA GeForce GTX 1660 Ti GPU (NVIDIA Corporation, Santa Clara, CA, USA), and 32 GB RAM, running Ubuntu 20.04 (Canonical Ltd., London, UK) with ROS Noetic. The results are summarized in Table 6.

As shown in Table 6, both MARVEL and ST-GICM achieve a 100% goal success rate in the representative ROS/Gazebo simulation runs, indicating that the generated high-level waypoints can be successfully executed by the local planner in the tested scenarios. ST-GICM shows a lower average high-level decision time than MARVEL, suggesting that the proposed decision module can be executed efficiently within the simulated navigation pipeline. Meanwhile, ST-GICM obtains higher final coverage, while requiring longer waypoint execution time, total runtime, and trajectory length. This trend is consistent with the observation that ST-GICM tends to sustain exploration progress rather than minimizing execution time alone. It should be noted that these results are obtained from representative ROS/Gazebo simulation scenarios and are intended to complement the main quantitative evaluation rather than serve as real-world robotic validation.

**Figure 8:**
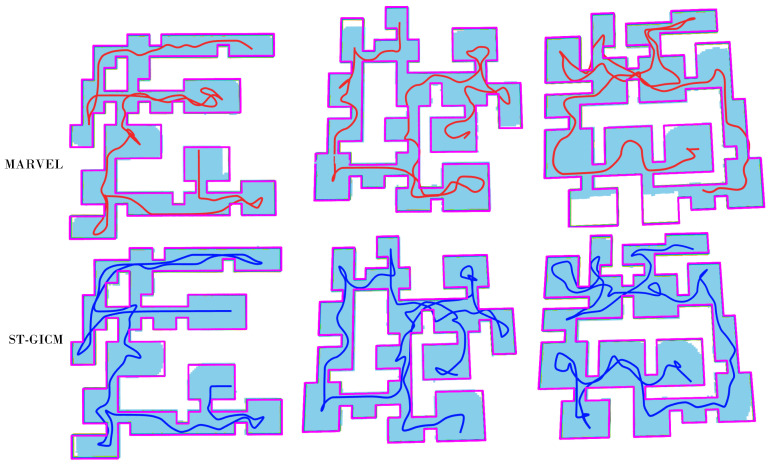
Qualitative comparison under ROS-based simulation. The different colors are used to distinguish the exploration trajectories generated by different methods.

As shown in Figure 8, a comparison of exploration trajectories in representative complex scenarios indicates that the baseline method MARVEL still exhibits evident local backtracking and repeated trials in narrow loops and highly similar regions. In contrast, ST-GICM advances in a more coherent manner and achieves broader coverage with fewer ineffective detours. This qualitative result is highly consistent with the conclusions in Table 3, particularly the lower repeated revisit rate and oscillation count of the proposed method, thereby demonstrating the superior behavioral stability of ST-GICM in a ROS-based closed-loop execution environment. These results indicate that ST-GICM can be integrated into a ROS-based closed-loop simulation pipeline. However, since the validation is still simulation-based, real-world deployment on physical robotic platforms remains future work.

## 6. Conclusions and Discussion

This paper proposes ST-GICM, a spatiotemporal graph learning framework with intrinsic curiosity for autonomous exploration. ST-GICM uses a dynamic viewpoint graph as the high-level decision carrier and integrates graph attention encoding, graph-level temporal fusion, and latent-prediction-based intrinsic rewards within a reinforcement learning framework. Experiments in simulated complex topological environments show that the proposed method achieves higher coverage, higher success rate, lower revisit ratio, and lower oscillation count than the compared baselines.

Further analysis indicates that the advantages of the proposed method stem from the deep coupling between exploration efficiency and behavioral robustness, rather than from merely shortening the trajectory length. Specifically, STRC enhances state discriminability through the integration of historical observations, thereby substantially suppressing redundant revisits, while IPM provides persistent intrinsic motivation when coverage gains begin to diminish. Although these experiments provide encouraging evidence for the effectiveness of ST-GICM in high-level graph-structured exploration and its compatibility with a simulated navigation pipeline, they do not constitute validation in fully realistic 3D environments or on physical robotic platforms. Future work will extend ST-GICM to realistic 3D exploration scenarios with richer sensing and motion constraints and further evaluate its performance on physical robotic systems.

## Figures and Tables

**Table 1:** Positioning of ST-GICM relative to representative exploration paradigms.

Method	Graph	Var. Action	Memory	Intrinsic	Topo. Curiosity	Limitation
Nearest Frontier	No	No	No	No	No	Myopic
NBVP	Sampling tree	Partial	No	No	No	Redundant paths
Utility-Weighted	No	No	No	No	No	Hand-crafted
MARVEL	Yes	Partial	Yes	No	No	Sparse rewards
ICM/RND-style methods	No	No	Optional	Yes	No	Non-topological
ST-GICM	Yes	Yes	STRC	IPM	Yes	This work

**Table 2:** Key implementation settings of ST-GICM.

Item	Setting
Graph encoder	6 GAT layers, 4 heads, 128 hidden dim, 512 FFN dim
Temporal memory	1-layer GRU, 256 hidden dim, sequence length 8
Policy and value	Pointer actor and twin Q critics
Intrinsic module	Forward and inverse MLP models, reward weight β=0.1
Optimization	Discrete SAC, Adam, batch size 32, replay buffer 10,000
Learning rates	Actor/critic $10^{-5}$, temperature/ICM $10^{-4}$
Training setup	Warm-up 2000 transitions, 4 updates per episode, target update every 64 updates
Training budget	1888 episodes, approximately 26.7 h
Training environments	Procedurally generated online during training

**Table 3:** Quantitative comparison of exploration performance in 100 test maps. Results are reported as mean ± standard deviation.

Method	Coverage (%)	Trajectory (m)	Success Rate (%)	Revisit Ratio ↓	Osc. Count ↓
Nearest Frontier	61.5±18.9	534.4±28.6	32	0.495±0.253	61.5±33.8
NBVP	74.7±6.4	1001.1±184.1	54	0.370±0.258	52.3±35.3
Utility-Weighted	68.9±6.7	619.9±36.2	38	0.419±0.269	56.8±34.5
MARVEL	80.1±16.8	758.3±238.6	62	0.161±0.195	11.8±24.8
**Ours (ST-GICM)**	92.7±6.3	759.66±43.45	**69**	0.097±0.050	5.3±4.7

Note: ↓ indicates that lower values are better; bold values indicate the best performance in each column.

**Table 4:** Ablation results on the full test set (mean±std).

Variant	Traj. Length (m)	Coverage@ $T_{\max}$	Dist@90	Success Rate (%)
Base (GAT)	681.27±106.88	0.803±0.169	589.89±51.42	22.0
+STRC	734.77±36.62	0.899±0.085	658.30±53.78	56.0
+IPM	646.90±26.49	0.844±0.080	566.78±59.26	26.0
**Ours**	759.66±43.45	0.927±0.063	676.80±76.05	**69.0**

Note: Bold values indicate the best performance in each column.

**Table 5:** Sensitivity analysis of key parameters. Results are reported as mean ± standard deviation over 30 test episodes.

Setting	Coverage (%)	Osc. Count	Decision Time (s)
β=0.0	90.51±7.52	5.27±2.57	0.1545±0.0096
β=0.05	91.79±7.20	5.20±2.47	0.1578±0.0114
β=0.10	91.74±7.55	5.23±2.58	0.1575±0.0107
β=0.20	91.01±7.31	5.00±2.14	0.1580±0.0109
$\delta_{g}=3.0$ m	92.50±6.37	5.07±2.34	0.1551±0.0103
$\delta_{g}=4.0$ m	91.74±7.55	5.23±2.58	0.1575±0.0107
$\delta_{g}=5.0$ m	91.12±6.94	5.53±2.45	0.1530±0.0111
$d_{h}=128$	90.43±7.85	6.83±5.22	0.1495±0.0100
$d_{h}=256$	91.74±7.55	5.23±2.58	0.1575±0.0107
$d_{h}=512$	91.69±6.77	5.47±2.12	0.1537±0.0094

**Table 6:** System-level indicators in representative ROS/Gazebo simulation runs. Results are reported as mean ± standard deviation over three scenarios.

Metric	MARVEL	ST-GICM
Total goals	124.33±3.21	117.00±6.08
Goal success rate (%)	100.00	100.00
Average high-level decision time (ms)	63.21±7.83	58.36±5.62
Mean waypoint execution time (s)	1.399±0.108	1.859±0.118
Total runtime (s)	180.74±12.41	224.94±18.36
Total trajectory length (m)	640.36±38.62	744.50±61.13
Final coverage (%)	84.32±5.05	87.77±9.39

## Data Availability

The data supporting the reported results are available from the corresponding author upon reasonable request.

## Abbreviations

The following abbreviations are used in this manuscript:

DRL	Deep Reinforcement Learning
GNN	Graph Neural Network
GAT	Graph Attention Network
SRE	Spatial Representation Encoder
STRC	Spatio-Temporal Reasoning Core
IPM	Intrinsic Prediction Module
SAC	Soft Actor-Critic
POMDP	Partially Observable Markov Decision Process
ESDF	Euclidean Signed Distance Field
RRT	Rapidly-exploring Random Tree
NBVP	Next-Best-View Planner
ROS	Robot Operating System
